# Transcellular transmission and molecular heterogeneity of aggregation-prone proteins in neurodegenerative diseases

**DOI:** 10.1016/j.mocell.2024.100089

**Published:** 2024-07-04

**Authors:** Eunmin Lee, Hyeonwoo Park, Sangjune Kim

**Affiliations:** Department of Biological Sciences and Biotechnology, Chungbuk National University, Cheongju, Chungbuk 28644, Korea

**Keywords:** Cell-to-cell transmission, Different strains, Neurodegenerative diseases, Protein aggregates, Selective vulnerability

## Abstract

The accumulation of aggregation-prone proteins in a specific neuronal population is a common feature of neurodegenerative diseases, which is correlated with the development of pathological lesions in diseased brains. The formation and progression of pathological protein aggregates in susceptible neurons induce cellular dysfunction, resulting in progressive degeneration. Moreover, recent evidence supports the notion that the cell-to-cell transmission of pathological protein aggregates may be involved in the onset and progression of many neurodegenerative diseases. Indeed, several studies have identified different pathological aggregate strains. Although how these different aggregate strains form remains unclear, a variety of biomolecular compositions or cross-seeding events promoted by the presence of other protein aggregates in the cellular environment may affect the formation of different strains of pathological aggregates, which in turn can influence complex pathologies in diseased brains. In this review, we summarize the recent results regarding cell-to-cell transmission and the molecular heterogeneity of pathological aggregate strains, raising key questions for future research directions.

## INTRODUCTION

Many neurodegenerative diseases are characterized by the pathological accumulation of specific disease-associated proteins. For example, in Alzheimer’s disease (AD), amyloid β (Aβ) and Tau protein aggregates are prominent (Baker et al., 1994, Braak and Braak, 1991); in Parkinson’s disease (PD), α-synuclein (α-syn) aggregates are prevalent (Braak et al., 2003, Del Tredici et al., 2002); TAR DNA-binding protein 43, superoxide dismutase 1, and fused in sarcoma aggregates have been implicated in amyotrophic lateral sclerosis (ALS) (Shaw and Eggett, 2000); and in Huntington’s disease (HD), mutant huntingtin has been identified (Graveland et al., 1985). These neurotoxic protein aggregates selectively target postmitotic neurons, as they cannot be diluted through cell division. Accumulating evidence suggests that protein aggregates induce dysfunction in a variety of cellular organelles, including the mitochondria and lysosomes (Burbidge et al., 2022, Lee et al., 2022, Pinho et al., 2014, Venkatesh et al., 2012, Wasilewski et al., 2017, Yuan et al., 2023), ultimately resulting in the progressive loss of neurons in the affected nerve tissue.

Intriguingly, both clinical and animal model studies have well-documented that misfolded pathological protein aggregates can spread throughout the brain in a prion-like manner (Angot et al., 2010, de Calignon et al., 2012, Jucker and Walker, 2013, Luk et al., 2012, Munch et al., 2011, Pecho-Vrieseling et al., 2014, Porta et al., 2018). Moreover, pathologies spreading between synaptically connected neurons have been shown to follow disease-specific distribution patterns of pathological proteins in the brains of patients (Braak et al., 2003, Kim et al., 2019). This transmission process involves the formation of pathological protein aggregates in donor cells, their transport at the synapse, and their uptake by recipient cells (Peng et al., 2020). These aggregates act as templates or seeds to recruit endogenous protein monomers and generate new pathological forms of diseased proteins (Guo and Lee, 2011, Luk et al., 2012, Mao et al., 2016). However, it is important to note that this transmission process is not observed in all synaptically connected regions (Surmeier et al., 2017), indicating that spreading pathologies are not strictly determined by synaptic networks alone. Recently, several studies have investigated the cellular and genetic factors that contribute to the selective neuronal vulnerability to pathological seeds. Additionally, researchers have focused more on understanding the nature of these pathological seeds to elucidate the transmission process as one of the complex pathologies of neurodegenerative diseases.

In this review, we summarize the recent experimental evidence regarding protein aggregation, cell-to-cell transmission, selective vulnerability, and molecular heterogeneity of pathological aggregates in the pathogenesis of neurodegenerative diseases. In doing so, we aim to discuss these findings and raise key questions to guide future research in this field.

### The Formation of Protein Aggregates

Proteins play crucial roles in nearly all cellular processes through the adoption of specific 3-dimensional conformations (Dobson, 2003). Molecular chaperones in the cytoplasm and endoplasmic reticulum promote proper protein folding. However, proteins containing intrinsically disordered regions are prone to aggregation, forming large protein aggregates and amyloids under physiological conditions (van der Lee et al., 2014). Amyloids have diverse functions, exhibiting multiple biological roles in vivo, including signal transmission, long-term synaptic modifications, and programmed cell death (Chiti and Dobson, 2006, Coustou et al., 1997, Li et al., 2012, Saupe, 2000, Si et al., 2003). However, extensive research has linked protein aggregation to numerous pathological conditions, including diabetes, as well as neurodegenerative disorders, such as AD and PD (Maji et al., 2009, Masino et al., 2011).

Several factors are known to promote protein aggregation. Genetic mutations in genes encoding diseased proteins can increase protein concentrations (Singleton et al., 2003) or destabilize the native form, thereby exposing segments of their structures externally and making them more prone to molecular interactions (Kurepa and Smalle, 2008). In addition, an age-dependent decline in cellular protein quality control and thermodynamic alterations in pH or temperature have been shown to promote amyloid formation in vivo (Higuchi-Sanabria et al., 2018). Importantly, the presence of homologous or heterologous seeds formed by amyloidogenic proteins significantly accelerates the rate of amyloid formation by aggregation-prone proteins (Walsh et al., 1999). Consequently, the formation and progression of pathological protein aggregates profoundly affect various cellular functions.

### The Pathological Mechanisms Underlying Protein Aggregation

Protein aggregation inhibits the ability of the native protein to perform its functions, resulting in cell dysfunction (Burre et al., 2010, Gauthier et al., 2004, Neumann et al., 2006, Trojanowski and Lee, 2005). As such, protein aggregates play pivotal roles in disease pathogenesis by disrupting cellular membranes and altering membrane conductivity (Brender et al., 2008, Kayed et al., 2004). Excessive amyloid accumulation can further impede mitochondrial adenosine triphosphate generation (Pinho et al., 2014, Venkatesh et al., 2012, Wasilewski et al., 2017) and compromise the ability of endoplasmic reticulum to detect misfolded proteins (Stojkovska et al., 2022). Furthermore, protein aggregates have been found to hinder lysosomal and proteasomal degradation pathways, resulting in the accumulation of misfolded proteins and formation of new aggregates (Burbidge et al., 2022, Lee et al., 2022, Yuan et al., 2023). Subsequently, lysosomal dysfunction causes cellular damage, including impaired chaperone-mediated autophagy, and defective mitochondrial accumulation (Bejarano and Cuervo, 2010, Martinez-Vicente et al., 2008). Moreover, excess amyloid-induced lysosomal rupture promotes the generation of reactive oxygen species via lysosomal enzymes (Freeman et al., 2013), while soluble N-ethylmaleimide-sensitive factor attachment protein receptor (SNARE) proteins further mediate lysosomal dysfunction (Cuddy et al., 2019). Mutant α-syn aggregates disrupt the trafficking of coat protein complex II vesicles from the rough endoplasmic reticulum to the cis-Golgi apparatus by impeding SNARE complex assembly (Thayanidhi et al., 2010). In addition to organelle dysfunction, protein aggregates can modulate the immune response within cells, thus influencing disease progression. In PD, neuroinflammation triggered by the cytokines TNF-α (tumor necrosis factor-alpha), IL-1β (interleukin-1 beta), and IL-6 (interleukin-6) promotes the formation of α-syn aggregates (Cheng et al., 2022). Notably, protein aggregates interact with other biomolecules, contributing to disease pathology through the formation of pathogenic entities, such as Lewy bodies (LBs) (Beyer and Ariza, 2007).

### Cell-to-Cell Transmission of Protein Aggregates

The lesions observed in many neurodegenerative disorders, including AD, PD, HD, and ALS, can be characterized by the accumulation of protein aggregates within various cellular compartments, including the nucleus and cytoplasm (Chiti and Dobson, 2006). Each of these conditions is characterized by the localization of distinct protein aggregates in specific brain regions, resulting in the selective degeneration of the neurons responsible for secreting particular neurotransmitters (Marambaud et al., 2009).

Several studies have previously linked the transmission of protein aggregates to prion-like mechanisms in many neurodegenerative diseases (Angot et al., 2010, de Calignon et al., 2012, Jucker and Walker, 2013, Luk et al., 2012, Munch et al., 2011, Pecho-Vrieseling et al., 2014, Porta et al., 2018). The term “prion-like” denotes the dissemination of protein aggregates that act as seeding agents. Initial findings revealed the formation of seeded aggregates of endogenous proteins in experiments where brain extracts from AD model primates were introduced into healthy primates, leading to the emergence of Aβ plaques (Baker et al., 1994). Similar phenomena were observed for Tau protein (Sanders et al., 2014) and α-syn protein (Luk et al., 2012), indicating that the mechanism is not limited to specific neurodegenerative diseases. Prion proteins are characterized by a high abundance of β-sheets, which influence their aggregation (Angot et al., 2010). Specifically, among the different protein aggregates prominent in neurodegenerative disorders, such as Aβ in AD and α-syn fibrils in PD, β-sheet dominance has been found to significantly impact prion-like spread (Conway et al., 2000, Jin et al., 2016). Several studies have revealed the presence of pathogenic proteins within the aggregates of healthy neurons transplanted into affected patients, underscoring the importance of investigating the effects of these transmission pathways (Angot et al., 2010, Li et al., 2008).

### Molecular Mechanisms Underlying the Cell-to-Cell Transmission of Protein Aggregates

**Fig. 1:**
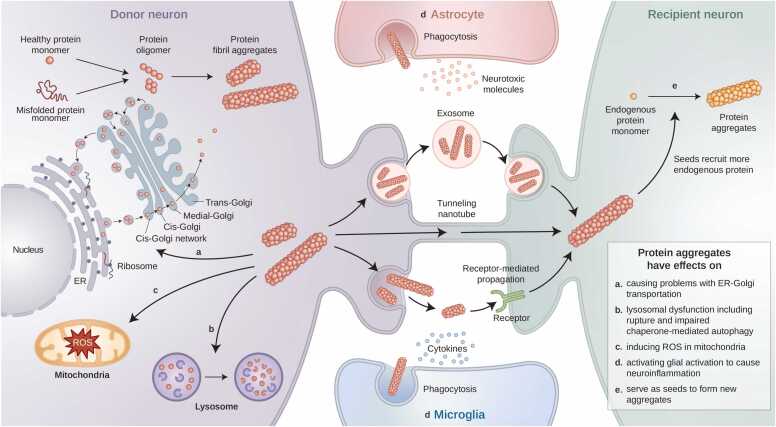
The effect of protein aggregates on cellular organelles and intercellular regions following cell-to-cell transmission is multifaceted. (a) Protein aggregates hinder the COPII-mediated transportation of proteins from the rough endoplasmic reticulum to the cis-Golgi, particularly in the context of mutations associated with protein aggregation. (b) Protein aggregates impede the delivery of chaperone-mediated autophagic targets to lysosomes. (c) Protein aggregates provoke lysosomal rupture, leading to oxidative stress and mitochondrial dysfunction. (d) Extracellular protein aggregates, transported via exosomes, tunneling nanotubes, and receptor-mediated propagation, etc., trigger neuroinflammation by modulating the reactive states of adjacent glial cells through cytokine induction. (e) Following cell-to-cell transmission, recipient neurons uptake the protein aggregates, which act as seeds to promote the aggregation of endogenous proteins, facilitating a prion-like spreading mechanism. COPII, coat protein complex II; ER, endoplasmic reticulum; ROS, reactive oxygen species. Illustrated images were generated using BioRender.com.

The formation of neuronal protein aggregates precedes their transmission to neighboring cells via extracellular release, followed by uptake and subsequent aggregate formation within recipient cells (Fig. 1). Notably, the protein aggregates implicated in neurodegenerative diseases are predominantly secreted through unconventional pathways, owing to the absence of conventional secretion signaling sequences (Tang, 2018). Extracellular vesicles, particularly exosomes (with a diameter of approximately 100 nm), represent a prominent secretory mechanism that facilitates intercellular communication and signal transduction by transporting cytokines, proteins, and nucleic acids (Thery, 2011). Indeed, studies have shown that glial cell-derived exosomes contribute to the degradation of Aβ by the exosome-associated insulin-degrading enzyme in models of AD (Bulloj et al., 2010, Tamboli et al., 2010). However, exosomes harbor proteins that are capable of forming aggregates and facilitating cell-to-cell transmission. The inhibition of exosome secretion by pharmacological agents attenuates the intercellular movement of pathogenic proteins, highlighting exosomes as potential therapeutic targets (Alvarez-Erviti et al., 2011, Dinkins et al., 2014). Tunneling nanotubes (TNTs) are slender intercellular structures, typically ranging from 50 to 200 nm in diameter, which emerge from filopodia-like protrusions primarily composed of actin filaments to extend toward adjacent cells (Rustom et al., 2004). Similar to exosomes, TNTs play crucial roles in intercellular communication and facilitate protein transport through TNT-like conduits (Sowinski et al., 2008). These structures have further been implicated in the pathology of AD and have been considered as promising targets for therapeutic intervention. Tau, which plays a pivotal role in AD pathology and helps to regulate microtubule stabilization within neurons, is a constituent of TNTs (Tardivel et al., 2016). Additionally, exogenous Tau promotes TNT formation, thus driving the intercellular transmission of protein aggregates (Tardivel et al., 2016). TNT-mediated trafficking is also observed in glial cells, where overloaded microglia with α-syn degradation machinery can transfer TNTs to neighboring microglia, thus favoring cell survival (Scheiblich et al., 2021).

The uptake of extracellular amyloids has been implicated in the initiation of intracellular aggregate formation, which subsequently leads to neurotoxicity and transmission (Friedrich et al., 2010, Hu et al., 2009, Jin et al., 2016). One such uptake mechanism is receptor-mediated endocytosis, while several membrane receptors have been identified in various neurodegenerative diseases. Lymphocyte-activation gene 3, a marker of CD4^+^ and CD8^+^ T cells (Huard et al., 1995), exhibits stronger binding affinity to α-syn fibrils than other monomers in the extracellular milieu. Lymphocyte-activation gene 3 facilitates the internalization of endogenous α-syn through endocytosis, promoting intracellular aggregation and neurodegeneration via transmission (Mao et al., 2016). Toll-like receptors (TLRs), another type of immune receptor, have also been extensively investigated for their role in protein aggregate transmission. TLR signaling is associated with inflammation, while TLR2 levels have been found to be elevated in PD lesions, thereby facilitating neuroinflammation through microglial activation and cytokine release (Dzamko et al., 2017). This receptor-mediated process facilitates protein trafficking between neurons, between neurons and glial cells (Kim et al., 2018), as well as between different glial cells (Giusti et al., 2024, Kim et al., 2013). Additionally, low-density lipoprotein receptor-related protein 1 (LRP1), which is crucial for lysosomal enzyme activity (Lillis et al., 2005), binds to Tau and influences its propagation (Rauch et al., 2020). The downregulation of LRP1 reduces Tau uptake and transneuronal spread of the pathology, suggesting that LRP1 is a potential target for tauopathy. Consequently, internalized protein aggregates serve as pathological seeds to recruit endogenous cognate proteins to form new pathological aggregates, thereby facilitating a new series of cell-to-cell transmission.

### Selective Vulnerability of Protein Aggregates

**Fig. 2:**
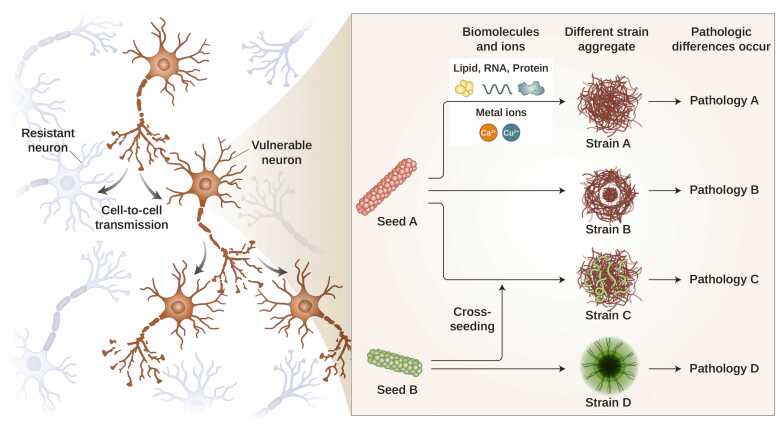
The molecular heterogeneity of pathogenic proteins contributes to the pathological diversity of neurodegenerative diseases. Seeds of pathogenic proteins are transmitted to synaptically connected neurons through cell-to-cell transmission. The different synaptically connected neurons are believed to exhibit differential vulnerability, with some neurons being selectively vulnerable (brown) while others (blue) to the transmission and aggregation of pathogenic proteins, which can lead to cell death. This phenomenon is thought to arise from environmental differences between cells. When seeds enter a cell, they are influenced by various biomolecules and ions, resulting in the formation of strains with distinct morphologies and aggregation patterns, or they may interact with each other to produce distinct morphologies. These different strains exhibit diverse morphological and biochemical features, which contribute to variations in clinicopathological characteristics, and ultimately result in different disease phenotypes. Illustrated images were generated using BioRender.com.

Intracellular protein aggregates can be transmitted between synaptically connected neurons. However, in many neurodegenerative diseases, aggregated pathogenic proteins are observed only in specific brain regions (Hass et al., 2021). This selective vulnerability observed in certain neuronal populations suggests that some neurons are more susceptible to the transmission and aggregation of pathogenic proteins, whereas others are resistant (Fig. 2) (Surmeier et al., 2017).

In AD, aggregates of the pathogenic protein Tau initially appear in the entorhinal olfactory cortex, and thereafter gradually spread to the hippocampus and neocortex (Braak and Braak, 1991). Selective neuronal degeneration caused by the vulnerability of the hippocampal and neocortical circuits in AD leads to a complete loss of cognition (Hof and Morrison, 2004). In PD, the pathogenic protein α-syn is aggregated in the olfactory system, substantia nigra, and cortex (Braak et al., 2003, Del Tredici et al., 2002); specifically, in the early PD stages, LBs or Lewy neurites are found in noncatecholaminergic neurons (Del Tredici et al., 2002). In HD, selective aggregation is observed in the medial spiny neurons of the striatum (Graveland et al., 1985), with huntingtin aggregates found in the striatum and the cerebral cortex (Coppen et al., 2018, McColgan et al., 2017). Particularly high rates of degeneration of corticostriatal connections have been reported in early HD; however, why this region is more vulnerable than other white matter connections remains unclear (McColgan et al., 2017).

The selective vulnerability of certain neuron populations is thought to be caused by differences in the composition of biomolecules in different intracellular environments (Fig. 2). Indeed, different neurodegenerative diseases differ in the composition of biomolecules in specific neuronal cell populations (Peng et al., 2018, Shaw and Eggett, 2000). The molecules targeting specific biomolecules include lipids and ions such as Ca^2+^, and it is thought that the interaction of these biomolecules with proteins can alter the degree of protein aggregation (Beasley et al., 2022, Beasley et al., 2021, Binolfi et al., 2008, Chen et al., 2023, Dou and Kurouski, 2022, Kurouski, 2023, Lautenschlager et al., 2018, Stonebraker et al., 2023, Zunke et al., 2018).

### Heterogeneity of Protein Aggregates Strains

It has been suggested that the many pathogenic proteins observed in neurodegenerative diseases exist in different strains with a variety of structural variants, which may result in different disease phenotypes (Dobson, 2003). These differences in strains may be attributed to disease duration, histopathological lesion profiles, and specific neuronal areas, and it is thought that the phenotypic traits of these strains persist upon serial transmission (Safar et al., 1998). As such, the pathological and clinical diversity of neurodegenerative diseases may be influenced by the variable seeding capacities and spreading behaviors of different strains of the same pathogenic protein (Aguzzi et al., 2007).

Although PD and multiple system atrophy are characterized by the accumulation of the same α-syn protein, they have different clinicopathological features. For example, α-syn aggregates from the cerebrospinal fluid of PD and multiple system atrophy analyzed by protein misfolding cyclic amplification technology were found to exist as different strains with distinct biochemical features and structural variants, demonstrating that different types of α-syn in synucleinopathies are responsible for the specificity of the disease phenotype (Morales et al., 2012, Shahnawaz et al., 2020, Strohaker et al., 2019). Similarly, when Tau strains isolated from patients with different tauopathies were analyzed, they were found to exhibit biochemically distinct morphologies and different phenotypic diversity (Sanders et al., 2014). Consequently, it is thought that different types of strains with the same protein have different morphological characteristics and virulence, ultimately leading to differences in the disease.

In copathologies, such as PD and dementia with Lewy bodies with AD, different disease protein aggregates occur simultaneously, including LB and Aβ plaque and neurofibrillary tangle formation (Galpern and Lang, 2006). One major protein has been shown to form amyloid aggregates by impairing the folding of other aggregation-prone proteins and promoting fibrillation. Specifically, different strains with different α-syn structures exhibit diverse cross-seeding abilities with Tau protein, resulting in pathological differences (Sanders et al., 2014). Differences in the presence and composition of the intracellular environment and biomolecules are thought to be responsible for these different strains. In fact, the protein-chameleon hypothesis has been proposed, suggesting that α-syn is characterized by conformational plasticity, able to select a series of conformations depending on the environment (Uversky, 2003). Consequently, it is thought that intracellular environmental differences and the presence of specific biomolecules can induce conformational modifications in proteins, leading to aggregation and accumulation, which are more likely to aggregate and metastasize in neuron cell populations with specific biomolecular compositions, resulting in the death of selective neuron populations.

Different strains of several proteins that cause neurodegenerative brain diseases are believed to affect cell-to-cell transmission. Differences in diffusion patterns have also been observed in strains of TAR DNA-binding protein 43, a known pathogenic protein in ALS that shows distinct characteristic neuropathological distributions (Porta et al., 2021). In addition, the heterogeneity of α-syn and Tau strains has been reported to influence seeding and propagation rates, leading to differences in pathogenesis (Lau et al., 2020). However, the role of the strain in the mechanisms of different cell-to-cell transmission pathways is not well understood, and more research is needed.

## CONCLUSION

During the past decade, substantial progress has been made in understanding the molecular mechanisms underlying transmission, particularly in regard to our understanding of the cell-to-cell transmission of pathological protein aggregates. However, several key gaps remain in our knowledge regarding why certain neuronal populations are susceptible to the transmission and aggregation of pathogenic proteins, while others are resistant. In addition to selective neuronal vulnerability, another major gap is in the characterization of the conformational diversity of pathological protein aggregates.

It is not yet known how cellular conditions promote the formation, progression, or amplification of pathological seeds in vivo. It may be necessary to characterize the molecular nature of pathological seeds through biophysical analysis or to dissect the composition of each amyloidogenic seed. To understand how these different strains are generated, further studies are required to experimentally mimic the nature of the different strains. Together with the protein misfolding cyclic amplification technique used to amplify small amounts of pathological proteins in diseased brains, these studies must address how the structural diversity of pathological seeds is reflected in the clinical and pathological diversity of neurodegenerative diseases. Finally, it is worth noting that copathologies of different pathological proteins often coincide in the aging brain. However, whether these diseases are caused by distinct pathogenic processes, an unintentional reaction to a shared trigger or the cross-seeding of one type of aggregated protein by another remains unclear and should be addressed in further studies.

Herein, we summarized the transcellular transmission process and molecular heterogeneity of pathological protein aggregates. Future research directions should include dissecting the precise descriptions and spreading behaviors of different pathological aggregate strains. These efforts will contribute to our understanding of the mechanisms underlying the transmission of pathogenic proteins and the complex pathologies of various neurodegenerative diseases.

## Author Contributions

S.K. conceptualized the scope of this review. E.L. and H.P. wrote the manuscript. E.L. and H.P. contributed to the figure production. All the authors have reviewed and approved the final manuscript.

## Declaration of Competing Interests

The authors declare that they have no known competing financial interests or personal relationships that could have appeared to influence the work reported in this paper.
